# Physical, Mechanical, and Adhesive Properties of Novel Self-Adhesive Resin Cement

**DOI:** 10.1155/2022/4475394

**Published:** 2022-04-08

**Authors:** Long Ling, Yumeng Ma, Yulin Chen, Raj Malyala

**Affiliations:** Glidewell Dental, Irvine, CA, USA

## Abstract

**Objective:**

To evaluate a newly developed self-adhesive resin cement on physical, mechanical, and adhesive properties and compare it with other commercial self-adhesive resin cements.

**Materials and Methods:**

Experimental self-adhesive resin cement (SARC) was formulated by our proprietary adhesive resin and filler technology. Maxcem Elite, RelyX Unicem 2, SpeedCem Plus, SmartCEM 2, and Calibra Universal 2 were selected for comparison. Working and setting times, film thickness, water sorption and solubility, flexural strength, and modulus were measured in accordance with ISO-4049. Consistency was tested according to modified ISO 4823. Shear bond strengths were conducted according to ISO 29022. The data were analyzed by one-way ANOVA and post hoc Tukey's tests (*p* ≤ 0.05).

**Results:**

All cements showed about 2–4 min working time and about 3–6 min setting time except that RelyX Unicem 2 has a longer working time (9'58”) and setting time (10'18”). All cements meet ISO standards for film thickness (≤50 µm), water sorption (≤40 µg/mm^3^) except Maxcem Elite (46.19 µg/mm^3^), and water solubility (≤7.5 µg/mm^3^) except SmartCEM 2 (11.35 µg/mm^3^) and Calibra Universal (9.87 µg/mm^3^). Experimental SARC showed significantly higher flexural strength and modulus than other cements (*p* < 0.001). For self-curing, Experimental SARC has statistically higher bond strength than other cements (*p* < 0.001) except statistically the same as RelyX Unicem 2 (*p* > 0.05). For light-curing, Experimental SARC showed significantly higher bond strength than other cements (*p* < 0.001) except statistically the same as Maxcem Elite and RelyX Unicem 2 (*p* > 0.05). For dual-curing, the bond strength of Experimental SARC is significantly higher than that of other cements (*p* < 0.001).

**Conclusion:**

The newly developed self-adhesive resin cement exhibited favorable bonding capability and physical and mechanical properties compared to other commercial self-adhesive resin cements and is a good option for cementation of indirect restorations with potential long-term clinical success.

## 1. Introduction

Cementation is a crucial step in indirect restoration such as crowns, inlays, onlays, and bridges. Cements play a significant role in cementation because long-term clinical success mainly depends on the cements used for adhesion between the tooth structure and the internal surface of the restoration [1–3]. Generally, the cements can be divided into two main categories: (1) water-based traditional cements such as zinc phosphate, polycarboxylate, glass ionomer, and resin-modified glass ionomer cement and (2) polymer-based resin cements. Compared with traditional cements, resin cements have provided some advantages over traditional cements, such as better esthetics, lower solubility, enhanced marginal integrity, high adhesion, and high mechanical properties [4–11], but conventional resin cements, including total-etch/esthetic and self-etch/adhesive resin cements, need etching, priming, and bonding prior to cementation. The multistep bonding procedure resulted in high technique sensitivity and postoperative sensitivity, especially for total-etch/esthetic resin cement.

Self-adhesive resin cement has been introduced and increasingly used during the last decade due to the ease of single-step cementation, reduced technique sensitivity, and lower postoperative sensitivity [1, 2, 11]. This new type of resin cement was developed to overcome some drawbacks of both traditional cements (such as zinc phosphate and glass ionomer) and conventional resin cements (such as total-etch resin cement). They combine the ease of handling of traditional cements with the favorable bonding strength, mechanical properties, and esthetics of conventional resin cements, resulting in a wide range of applications. Self-adhesive resin cement simplified the bonding procedure with the introduction of acidic monomers in their composition without separate use of etchant and/or primer/adhesive. These acidic monomers with carboxylic or phosphoric acid groups are used to achieve demineralization and infiltration of the tooth structures (enamel and dentin), resulting in micromechanical retention and additional chemical attachment on the tooth structure [6, 12, 13]. Since the cement is much more viscous and acidic monomer concentration is much lower as compared with the etchant and primer/adhesive used by conventional resin cements, this infiltration into the tooth structure is limited [14]. Generally, self-adhesive resin cements are not strong as conventional resin cements on bond strength, especially for enamel bonding. Therefore, self-adhesive resin cement with improved bond strength is always highly desirable without sacrificing physical and mechanical properties.

The purpose of this study is to evaluate a newly developed self-adhesive resin cement and compare it with other commercial self-adhesive resin cements. The hypothesis is that this new self-adhesive resin cement has improved physical, mechanical, and bonding properties compared to commercial self-adhesive resin cements.

## 2. Materials and Methods

### 2.1. Materials

Experimental self-adhesive resin cement (Experimental SARC or Exp) was formulated by our proprietary adhesive resin and filler technology, which included acidic monomer, non-acidic monomers, dual-cured initiator systems, inhibitor, and fillers. 10-methacryloyloxydecyl dihydrogen phosphate (10-MDP) as an acidic monomer and bisphenol a diglycidyl ether dimethacrylate (BisGMA) as rigid space-filling monomers with other dental monomers such as urethane dimethacrylate were used for resin system. Dual-curing initiator systems were composed of cumene hydroperoxide/(2, 3-difluorophenyl)thiourea as redox initiator system for self-curing and camphorquinone, bis(2, 4, 6-trimethylbenzoyl)-phenyl-phosphineoxide, and ethyl 4-dimethylaminobenzoate as photo initiators for light-curing. 2, 6-di-(tert-butyl)-4-methylphenol was used as inhibitor. Fillers consisted of barium boron fluoroaluminosilicate glass, OX-50 fumed silica, and ytterbium fluoride. The homogeneous resin mixtures were first obtained by stirring resin monomers with the additives until dissolved. The resulting resin mixtures were further mixed with fillers until a uniform flowable paste was formed. Five commercially available self-adhesive resin cements, including Maxcem Elite (Kerr, Orange, CA), RelyX Unicem 2 (3M ESPE, St. Paul, MN), SpeedCem Plus (Ivoclar Vivadent, Schaan, Liechtenstein), SmartCEM 2 (Dentsply Sirona, York, PA), and Calibra Universal (Dentsply Sirona, York, PA), were selected in this study for comparison. Further information about these self-adhesive resin cements in this study are shown in Table 1.

### 2.2. Working Time and Setting Time

Working time and setting time (*n* = 5) at 23°C were determined using thermocouple apparatus (UTC-USB, Omega Engineering Inc., Norwalk, CT) described in ISO 4049 (2009).

### 2.3. Consistency

The consistency was determined according to the modified method provided in ISO 4823 (1992) as modified for elastomeric impression materials. A mixed cement (0.3 ± 0.01 g) was placed between the glass plates, and a standard weight of 120 g was applied to the cement for 1 minute. The average value was obtained based on the maximum and minimum diameters of the disk. Three measurements were made for each cement.

### 2.4. Film Thickness

Film thickness was measured in accordance with ISO 4049 (2009). Mixed cement was placed between two glass plates of uniform thickness (5 mm) and loaded under 150 N for 10 minutes. Film thickness was determined by the thickness difference of the glass plates with and without the cement film (*n* = 5).

### 2.5. Water Sorption and Solubility

The water sorption and solubility tests were conducted according to ISO 4049 (2009). The disk specimens ((15.0 ± 0.1) mm in diameter and (1.0 ± 0.1) mm thickness; *n* = 5) were prepared in a Teflon mold and light-cured with a light intensity of approximately 1,000 mW/cm^2^ (Bluephase Style, Ivoclar Vivadent AG, Schaan, Liechtenstein) for 20 × 5 seconds (initially on the center and then on top, bottom, left, and right of the specimen). The specimens were stored in a desiccator at 37°C for 22 h, transferred to another desiccator at 23°C for 2 h and weighed until a constant mass (*m*_1_) was obtained by repeating this cycle. The specimens were then stored in deionized water at 37°C for 7 days. The surface water on the specimen was blotted away free from visible moisture and waved in the air for 15 s. Then the mass *m*_2_ of specimens was recorded. The specimens were reconditioned in a desiccator and weighed until a constant weight (*m*^3^) was obtained using the cycle described above.

Water sorption:

(1) $$\begin{array}{c} W_{sp}=\frac{m_{2}-m_{3}}{V}. \end{array}$$

Solubility:

(2) $$\begin{array}{c} W_{s1}=\frac{m_{1}-m_{3}}{V}, \end{array}$$

where V is the volume of the specimen.

### 2.6. Flexural Strength and Flexural Modulus

Flexural strength and modulus were determined by the three-point bending method in accordance with ISO-4049 (2009). The bar-shaped specimens (thickness × width × length = 2 × 2 × 25 mm; *n* = 5) were prepared from cement materials (self-cured for 15 min at 37°C and stored in DI water at 37 °C for 24 hours) and tested under the crosshead speed of 0.75 mm/min by using a test fixture with a 20 mm support span on an Instron 5564 universal testing machine. Flexural modulus was determined from the slope of the linear region of the stress-strain curve. The following formulas were used to calculate the flexural strength and modulus:

For flexural strength (FS),

(3) $$\begin{array}{c} \sigma_{f}=\frac{3Pl}{2wb^{2}}. \end{array}$$

For flexural modulus (FM),

(4) $$\begin{array}{c} E_{f}=\frac{sl^{3}}{4wb^{3}}, \end{array}$$

where *P* is the maximum load (N), *l* is the test span (mm), *w* is the width of the specimen (mm), *b* is the thickness (mm) of the specimen, and *s* is the slope of the linear portion of the stress-strain curve (N/mm).

### 2.7. Shear Bond Strength

Specimen preparation and testing were conducted according to ISO 29022 (notched-edge shear bond strength test; 2013). Caries-free human molar teeth (this study did not involve human participants; the teeth could not be connected to the patient from which they were extracted; and all testing were performed at Glidewell Laboratories; therefore, this study was exempt from IRB review and approval) were cut (buccolingual section), embedded in acrylic resin mixed with powder and wet polished sequentially with 500 and 1,200 grit SIC on Grinder-Polisher (EcoMet 300 Pro, Buehler, Lake Bluff, IL). The cements were placed on a polished tooth surface (dentine) using an Ultradent jig mold and self-cured (for 15 minutes at 37°C), light-cured (for 20 seconds with a light intensity of approximately 1,000 mW/cm^2^) and dual-cured (self-cured for 5 minutes at 37°C, followed by light-cured for 20 seconds), respectively. Bonded specimens were stored in DI water at 37^o^C for 24 hours and tested for shear bond strength to dentin at a crosshead speed of 1.0 mm/min until failure on an Instron 5564 universal testing machine (*n* = 12).

### 2.8. Statistical Analysis

Statistical analysis was performed using Minitab 18 Statistical Software (Minitab, LLC, State College, PA, USA). The results for each mechanical and bonding property were analyzed by one-way ANOVA and Tukey's post hoc comparison. The significance level was set at *α* = 0.05. Normality and homogeneity of all testing data were evaluated before ANOVA. Normality was first conducted using Anderson–Darling test, which showed that all groups are normal distribution (*p* > 0.05) except light-cured bond strength of RelyX Unicem 2 (*p* < 0.05). For homogeneity evaluation, the Bartlett test was used for each property with normal distribution, and the Levene test was used for light-cured bond strength with non-normal distribution due to RelyX Unicem 2. Both the Bartlett test and the Levene test showed homogeneity of variance between the groups of each property (*p* > 0.05).

## 3. Results

**Figure 1:**
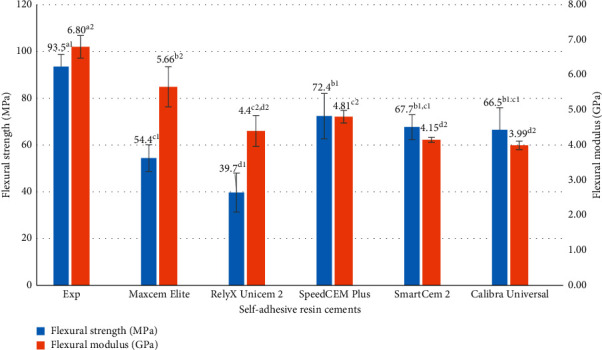
Flexural strength and modulus of self-adhesive resin cements (values with the same superscript are statistically equivalent between the tested groups).

**Figure 2:**
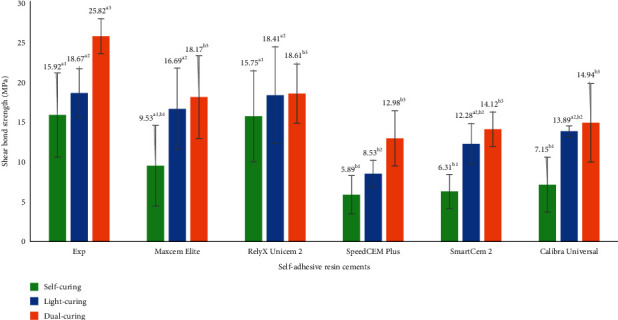
Shear bond strength to dentin of self-adhesive resin cements (values with the same superscript are statistically equivalent between the tested groups).

The test results for physical, mechanical, and adhesive properties of experimental and commercially available self-adhesive resin cements, which include working time and setting time, consistency, film thickness, water sorption and solubility, flexural strength, flexural modulus, and shear bond strength, are shown in Table 2 and Figures 1 and 2. The data were analyzed by one-way ANOVA and Tukey's tests (*p* ≤ 0.05). Values with the same superscript are statistically equivalent between the tested groups in Figures 1 and 2 according to the statistical tests used.

All tested self-adhesive resin cements showed suitable working time (about 2–4 min) and setting time (about 3–6 min), which meet the ISO 4049 requirement, except that RelyX Unicem 2 has a longer working time (9'58”) and setting time (10'18”). Experimental SARC has almost the same consistency as Maxcem Elite, SpeedCem Plus, and SmartCEM 2 but slightly lower than RelyX Unicem 2 and Calibra Universal (Table 2). The film thickness of all cements meets the ISO standard (≤50 µm). Experimental SARC showed lower film thickness than Maxcem Elite, SpeedCem Plus, and SmartCEM 2, similar to Calibra Universal and RelyX Unicem 2 (Table 2). The water sorption of all cements meets the ISO standard (≤40 µg/mm^3^) except Maxcem Elite (46.19 µg/mm^3^), Experimental SARC has the lowest value (25.91 µg/mm^3^) among all cements. Experimental SARC has lower water solubility than Maxcem Elite, SmartCEM 2, and Calibra Universal. RelyX Unicem 2 showed a negative value of water solubility. For mechanical properties, Figure 1 showed that Experimental SARC had statistically higher flexural strength and flexural modulus than other cements (*p* < 0.001).

Shear bond strengths to dentin were evaluated by self-curing, light-curing, and dual-curing (Figure 2). For self-curing, Experimental SARC has statistically higher bond strength than other cements (*p* < 0.001) except statistically the same as RelyX Unicem 2 (*p* > 0.05). For light-curing, Experimental SARC showed significantly higher bond strength than other cements (*p* < 0.001) except statistically the same as Maxcem Elite and RelyX Unicem 2 (*p* > 0.05). For dual-curing, the bond strength of Experimental SARC is significantly higher than that of other cements (*p* < 0.001).

## 4. Discussion

For most indirect restorations such as crowns, bridges, inlays, and onlays, cementation was achieved mainly by self-curing reaction (autopolymerization) as little or no light is transmittable through the restorative materials. The International Standards Organization (ISO) sets a minimum working time of 90 seconds and a maximum setting time of 10 minutes. A working time of less than 90 seconds may lead to difficulties in clinical use. In general, a working time of a couple of minutes would be appropriate for clinicians to have enough time for manipulation during the luting process in clinical practice. Once the working time is passed, it is advantageous to have the final set followed rapidly. Considering the clinical application of resin cement luting systems, a setting time of over 10 minutes is an undesirably long time for a luting cement to obtain an optimal setting characteristic without compromising the margin integrity [15]. All self-adhesive resin cements tested passed according to the standard except that RelyX Unicem 2 has the longest working time (9'58”) and setting time (10'18”). Working time and setting time were controlled by the resin cement's composition. Manufacturers set their appropriate working time and setting time in compliance with the ISO standard based on their proprietary cement composition.

Consistency and film thickness are clinically relevant and essential for the clinician to consider the manipulation and clinical success during the luting procedure in restoration [16]. Consistency represents the flowability and mainly affects the handling property of the cement. Five commercially available self-adhesive resin cements used in this study showed the consistency of 2.70 to 2.90 cm, Experimental SARC has the same or similar consistency as these commercially available self-adhesive resin cements. Film thickness varies greatly among the cements. A low film thickness can improve restoration seating and decrease marginal leakage and loss of marginal integrity, which will reduce plaque accumulation, periodontal disease, and secondary caries [17, 18]. Cement film thickness is affected by multiple factors such as consistency, filler content, resin composition, and the degree of polymerization [16,19,20]. All cements were below the maximum limit of 50 µm ISO sets. Experimental SARC showed lower film thickness than Maxcem Elite, SpeedCem Plus, and SmartCEM 2 and similar film thickness to RelyX Unicem 2 and Calibra Universal.

The water sorption and solubility of a cement play an important role in the life time of the cement for an indirect restoration. Water sorption and solubility can affect the restoration's retention, strength, biocompatibility, dimensional stability, microleakage, secondary caries, and the like [9, 21–24]; for example, in the oral environment, the restoration becomes more sensitive to moisture that may increase the potential of bond degradation and cement dissolution at the marginal gap, which may result in weakening and fracture of the indirect restoration [23]. The International Standards Organization sets maximum water sorption of 40 µg/mm^3^ and a maximum solubility of 7.5 µg/mm^3^. According to ISO Standards, all tested cements passed water sorption (≤40 µg/mm^3^) except Maxcem Elite (46.19 µg/mm^3^). All tested cements passed water solubility (≤7.5 µg/mm^3^) except SmartCEM 2 (11.35 µg/mm^3^) and Calibra Universal (9.87 µg/mm^3^). Experimental SARC had the lowest water sorption (25.91 µg/mm^3^) among all cements and lower water solubility than Maxcem Elite, SmartCEM 2, and Calibra Universal while higher than RelyX Unicem 2 (negative value) and SpeedCem Plus. These different values of water sorption and solubility are mainly attributed to the cement composition, primarily the chemical composition of resin matrices as these cements have similar filler loading (about 70 wt.%) [5, 22, 25, 26]. The hydrophilic components such as acidic monomer and hydrophilic monomer and crosslinking density have a crucial effect on water sorption and solubility properties [22, 26–28]. Experimental SARC and SpeedCem Plus used 10-methacryloyloxydecyl dihydrogen phosphate (10-MDP) as self-etch and adhesive monomer in their resin composition, while Maxcem Elite, SmartCEM 2, and Calibra Universal used glycerol phosphate dimethacrylate (GPDM), dipentaerythritol pentaacrylate monophosphate (PENTA) and 4-methacryloxyethyl trimellitate anhydride (4-META), respectively (Table 1). The longer ethylene chain of 10-MDP makes this monomer more hydrophobic than GPDM, PENTA, and 4-META [29]. This is probably one of the reasons that Experimental SARC and SpeedCem Plus exhibited lower water sorption and solubility than Maxcem Elite, SmartCEM 2, and Calibra Universal. RelyX Unicem 2 showed a negative value of water solubility; it does not mean some components (such as unreacted monomers) of RelyX Unicem 2 did not dissolve. The possible reason is that the absorbed water by the cement is bound into the polymer network and cannot be reversely extracted, leading to negative solubility [22, 30, 31].

Flexural strength is the ability of a material to resist deformation under load, which combines compressive stress and tensile stress. Flexural modulus is a measure of a material's stiffness when flexed. The mechanical properties of all cements were assessed by flexural strength and modulus in self-cure mode as flexural properties are very important mechanical properties for composite materials and the cementation mainly depends on the self-curing process. Adequate flexural strength and modulus can transit or adjust the stress between the restorations and tooth structure without fracture and/or permanent deformation; this will increase the failure resistance of the cemented restoration under applied forces and protect the brittle restoration materials [17, 32]. Experimental SARC exhibited statistically higher flexural strength and flexural modulus than other cements. Like the physical properties mentioned above, different flexural strength and modulus resulted mainly from the differences in cement composition (resins and fillers) between Experimental SARC and other cement materials. Experimental SARC was formulated by our proprietary resin and filler technology for our future product, which was based on the consideration of the cement strength and other properties; for example, Experimental SARC introduced bisphenol A glycidyl methacrylate (BisGMA) as a rigid space-filling monomer to increase the strength and stiffness, while other cements did not have such monomer as bisphenol A glycidyl methacrylate (BisGMA) based on the limited information provided by the manufacturers. It seems that SpeedCem Plus used some monomers the same as Experimental SARC, for example, urethane dimethacrylate (UDMA) and triethyleneglycol dimethacrylate (TEGDMA). But SpeedCem Plus does not have bisphenol A glycidyl methacrylate (BisGMA). In addition, besides TEGDMA, SpeedCem Plus contains another two flexible long-chain monomers that are polyethylene glycol dimethacrylate (PEGDMA) and 1,10-decandiol dimethacrylate (DDDMA). This is probably why SpeedCem Plus showed significantly lower flexural strength and modulus than Experimental SARC even it has a little higher filler loading (base: 75 wt.% and catalyst: 69.8 wt.%) than Experimental SARC (70 wt.%). RelyX Unicem 2 had the lowest flexural strength. This is probably because RelyX Unicem 2 contains calcium hydroxide in its formulation, which is the only cement claimed by the manufacturer [6]. Calcium hydroxide has an adverse effect on mechanical properties and decreases the mechanical strength of resin-based materials [31, 33].

Generally, self-adhesive resin cements showed favorable bond strength on dentin and lower bond strength on enamel surfaces compared to conventional resin cements [34–37]. Unlike conventional resin cements using separate etchants and/or primers/adhesives, the etching and adhesive properties of self-adhesive resin cements are from the presence of acidic monomers in their composition. The structure and concentration of the acidic monomers in cement formulation have a crucial impact on the bonding strength to substrates [13, 38, 39]. Shear bond strength to dentin of dual-cured self-adhesive resin cements in this study was assessed by self-curing, light-curing, and dual-curing. There are statistically significant differences in bond strength to dentin among these cements. Experimental SARC showed statistically higher or higher bond strengths than most of the other cements for self-curing, light-curing, and dual-curing modes. The findings are related to resin and filler compositions, especially acidic monomers (structure and concentration) [13, 38, 39]. The acidic monomers and other hydrophilic components have a low pH value and hydrophilic properties at the initial setting to facilitate good wetting and bonding to the tooth structure. The inorganic fillers and hydroxyapatite in the teeth provide the neutralization function to the initial low pH of the cement by the reaction with acidic monomers. The adhesive properties and pH neutralization vary significantly among the self-adhesive resin cements [16, 37, 40]; therefore, these self-adhesive cements exhibited different bond strengths. The limited information provided by the manufacturers showed that most tested cements used different phosphate acidic monomers as mentioned above. The acidic monomer in Experimental SARC is 10-methacryloyloxydecyl dihydrogen phosphate (10-MDP), which has a longer ethylene chain and is more hydrophobic than other phosphoric acid monomers such as glycerol phosphate dimethacrylate (GPDM) and dipentaerythritol pentaacrylate monophosphate (PENTA) [29]. This hydrophobicity of the longer ethylene backbone enhanced the pH neutralization capacity of the cement, which resulted in less susceptible to hydrolysis over time and greater bonding than other self-adhesive resin cements with different phosphate monomers [39, 41, 42]. Although it used the same acidic monomer (10-methacryloyloxydecyl dihydrogen phosphate (10-MDP)) as Experimental SARC used, SpeedCem Plus had significantly lower bond strength than Experimental SARC because the bond strength depends on not only the acidic monomer and its concentration but also other monomers and initiator systems used. Unfortunately, like other commercial self-adhesive resin cements, it is difficult to obtain accurate information of SpeedCem Plus about its resin composition. It is only assumed here that SpeedCem Plus has a low concentration of 10-MDP, or other monomers may not promote the bonding process with 10-MDP.

In addition, for each cement, the bond strength in the light-curing mode is higher than the corresponding self-curing mode, and the dual-curing mode produced a higher bond strength than the respective self-curing and light-curing. This is probably that light curing can provide a stronger energetic curing condition in this study. Clearly, dual-curing leads to higher bond strength due to more energetic curing conditions and a high degree of polymerization compared to individual self-curing or light curing. These results are consistent with other studies reported [43, 44], indicating that dual-curing (self-curing followed by additional light-curing) is highly recommended for adhesive cementation of indirect restorations.

## 5. Conclusion

The hypothesis has been proven, that is, a newly developed self-adhesive resin cement exhibited favorable bonding capability and physical and mechanical properties compared to other commercial self-adhesive resin cements based on the findings of this study for shear bond strength, working time and setting time, consistency, film thickness, water sorption and solubility, flexural strength, and flexural modulus. Experimental self-adhesive resin cement is a good alternative with a simplified clinic bonding procedure for cementation of indirect restoration.

## Figures and Tables

**Table 1:** Self-adhesive resin cements used in this study.

Material	Manufacturer	Resins	Fillers	Filler content (wt.%)
Exp. SARC	Glidewell	BisGMA, UDMA, TEGDMA, MDP, initiators, and inhibitor	Barium boron fluoroaluminosilicate glass, fumed silica, and ytterbium fluoride	70
MaxCem Elite	Kerr	Methacrylate esters, GPDM, HEMA, activators, and stabilizers	Mineral fillers and ytterbium fluoride	69
RelyX Unicem 2	3M ESPE	Methacrylated phosphoric esters, dimethacrylate, acetate, initiators, and stabilizers	Glass fillers, silica, and calcium hydroxide	70
SpeedCEM Plus	Ivoclar Vivadent	UDMA, TEGDMA, PEGDMA, DDDMA, MDP, dibenzoyl peroxide, and stabilizer	Barium glass and silica ytterbium trifluoride	75 (Base)/69.8 (Cat)
SmartCEM 2	Dentsply Sirona	UDMA, EBPADMA, di- and tri-functional function diluents, PENTA, 4-META,initiators, accelerators, and stabilizer	Barium boron fluoroaluminosilicate glass and amorphous silicon dioxide	69
Calibra Universal	Dentsply Sirona	UDMA, di- and tri-methacrylate, phosphoric acid modified acrylate, initiators, accelerators, stabilizer, and BHT	Barium boron fluoroaluminosilicate and amorphous silicon dioxide	73

BisGMA, bisphenol a diglycidyl ether dimethacrylate, UDMA, urethane dimethacrylate, TEGDMA, triethyleneglycol dimethacrylate, MDP, methacryloyloxydecyl dihydrogenphosphate, GPDM, glycerol phosphate dimethacrylate, HEMA, 2-hydroxyethyl methacrylate, PEGDMA, polyethylene glycol dimethacrylate, DDDMA, 1,10-decanediol dimethacrylate, EBPADMA, ethoxylated Bisphenol a dimethacrylate, PENTA, dipentaerythritol pentaacrylate monophosphate, 4-META, 4-methacryloxyethyl trimellitate anhydride, and BHT, butylated hydroxytoluene. The composition of the resins and fillers was obtained from the manufacturers.

**Table 2:** Physical properties of self-adhesive resin cements.

Properties\Cements	Exp	MaxCem Elite	RelyX Unicem 2	SpeedCEM Plus	SmartCEM 2	Calibra Universal
Working time (min. sec.)	1'53” ± 12”	1'53” ± 19”	9'58” ±18”	4'8” ± 23”	2'15” ± 26”	2'07” ± 14”
Setting time (min. sec.)	3'37” ± 15”	3'23” ± 13”	10'18” ± 17”	6'33” ± 56”	3'48” ± 30”	3'42” ± 11”
Consistency (cm)	2.71 ± 0.02	2.73 ± 0.0	2.80 ± 0.0	2.70 ± 0.0	2.70 ± 0.0	2.87 ± 0.04
Thickness (µm)	23.7 ± 3.2	37.6 ± 8.7	20.6 ± 4.1	33.8 ± 5.3	34.4 ± 3.7	22.2 ± 2.3
Water sorption (µg/mm^3^)	25.91 ± 0.64	46.19 ± 2.69	34.54 ± 0.96	30.74 ± 2.40	38.65 ± 1.33	38.34 ± 1.33
Solubility (µg/mm^3^)	2.94 ± 0.94	7.06 ± 1.87	-1.93 ± 1.12	0.43 ± 1.38	11.35 ± 0.58	9.87 ± 0.85

## Data Availability

The data used to support the findings of this study are available from the corresponding author upon request.
